# Synthesis of Electrospun TiO_2_ Nanofibers and Characterization of Their Antibacterial and Antibiofilm Potential against Gram-Positive and Gram-Negative Bacteria

**DOI:** 10.3390/antibiotics9090572

**Published:** 2020-09-03

**Authors:** Mohammad Azam Ansari, Hani Manssor Albetran, Muidh Hamed Alheshibri, Abdelmajid Timoumi, Norah Abdullah Algarou, Sultan Akhtar, Yassine Slimani, Munirah Abdullah Almessiere, Fatimah Saad Alahmari, Abdulhadi Baykal, It-Meng Low

**Affiliations:** 1 Department of Epidemic Disease Research, Institute for Research & Medical Consultations (IRMC), Imam Abdulrahman Bin Faisal University, P.O. Box 1982, Dammam 31441, Saudi Arabia; maansari@iau.edu.sa; 2 Department of Basic Sciences, College of Education, Imam Abdulrahman Bin Faisal University, P.O. Box 2375, Dammam 31451, Saudi Arabia; halbatran@iau.edu.sa; 3 Basic Science Department, Deanship of Preparatory Year and Supporting Studies & Basic and Applied Scientific Research Center, Imam Abdulrahman Bin Faisal University, P.O. Box 1982, Dammam 31441, Saudi Arabia; mhalheshibri@iau.edu.sa; 4 Physics Department, Faculty of Applied Science, Umm AL-Qura University, Makkah 24231, Saudi Arabia; timoumiabdelmajid@yahoo.fr; 5 Department of Biophysics, Institute for Research & Medical Consultations (IRMC), Imam Abdulrahman Bin Faisal University, P.O. Box 1982, Dammam 31441, Saudi Arabia; nalgarou@iau.edu.sa (N.A.A.); suakhtar@iau.edu.sa (S.A.); yaslimani@iau.edu.sa (Y.S.); malmessiere@iau.edu.sa (M.A.A.); 6 Department of Physics, College of Science, Imam Abdulrahman Bin Faisal University, P.O. Box 1982, Dammam 31441, Saudi Arabia; 7 Department of Nano-Medicine Research, Institute for Research & Medical Consultations (IRMC), Imam Abdulrahman Bin Faisal University, P.O. Box 1982, Dammam 31441, Saudi Arabia; fsalahmari@iau.edu.sa (F.S.A.); abaykal@iau.edu.sa (A.B.); 8 Department of Physics and Astronomy, Curtin University, GPO Box U1987, Perth, WA 6845, Australia

**Keywords:** TiO_2_ nanofibers, electrospinning, biofilm prevention and control, multidrug-resistant bacteria, biomedical application

## Abstract

Recently, titanium dioxide (TiO_2_) nanomaterials have gained increased attention because of their cost-effective, safe, stable, non-toxic, non-carcinogenic, photocatalytic, bactericidal, biomedical, industrial and waste-water treatment applications. The aim of the present work is the synthesis of electrospun TiO_2_ nanofibers (NFs) in the presence of different amounts of air–argon mixtures using sol-gel and electrospinning approaches. The physicochemical properties of the synthesized NFs were examined by scanning and transmission electron microscopies (SEM and TEM) coupled with energy-dispersive X-ray spectroscopy (EDX), ultraviolet-visible spectroscopy and thermogravimetric analyzer (TGA). The antibacterial and antibiofilm activity of synthesized NFs against Gram-negative *Pseudomonas aeruginosa* and Gram-positive methicillin-resistant *Staphylococcus* *aureus* (MRSA) was investigated by determining their minimum bacteriostatic and bactericidal values. The topological and morphological alteration caused by TiO_2_ NFs in bacterial cells was further analyzed by SEM. TiO_2_ NFs that were calcined in a 25% air-75% argon mixture showed maximum antibacterial and antibiofilm activities. The minimum inhibitory concentration (MIC)/minimum bactericidal concentration (MBC) value of TiO_2_ NFs against *P. aeruginosa* was 3 and 6 mg/mL and that for MRSA was 6 and 12 mg/mL, respectively. The MIC/MBC and SEM results show that TiO_2_ NFs were more active against Gram-negative *P. aeruginosa* cells than Gram-positive *S. aureus*. The inhibition of biofilm formation by TiO_2_ NFs was investigated quantitatively by tissue culture plate method using crystal violet assay and it was found that TiO_2_ NFs inhibited biofilm formation by MRSA and *P. aeruginosa* in a dose-dependent manner. TiO_2_ NFs calcined in a 25% air-75% argon mixture exhibited maximum biofilm formation inhibition of 75.2% for MRSA and 72.3% for *P. aeruginosa* at 2 mg/mL, respectively. The antibacterial and antibiofilm results suggest that TiO_2_ NFs can be used to coat various inanimate objects, in food packaging and in waste-water treatment and purification to prevent bacterial growth and biofilm formation.

## 1. Introduction

Titanium dioxide (TiO_2_) is among the investigated photocatalytic nanomaterials and is used extensively in diverse applications and for diverse purposes [1]. TiO_2_ nanomaterials are widely used in waste-water treatment and purification, air-pollutant decomposition, implantable devices, air-conditioning filters, hydrophilic coatings, self-cleaning and self-disinfecting devices, pesticide degradation (e.g., herbicides, insecticides and fungicides) and in the production of hydrogen fuel [2,3]. TiO_2_ is usually non-toxic, highly durable with a high refractive index, high absorption of light and a lower-cost production with antibacterial activity [4,5]. Because of its strong stability, TiO_2_ materials can be applied easily on inanimate items, e.g., metal, glass and biomedical implants [5]. Recently, TiO_2_ nanoparticles (NPs) have attracted increased interest in the scientific and industrial community because of their extensive applications in biological and pharmaceutical areas, purification of environmental sources, electronic system, solar energy cells, photocatalysts, photo-electrodes and gas sensors. TiO_2_ NPs are proven to be employed in food technology, drugs, cosmetics, paint pigment, ointments and toothpaste [6,7]. Because of their cost-effective, safe, stable, non-toxic, non-carcinogenic, photo-induced super-hydrophobicity and antifogging properties, TiO_2_ NPs have been used to kill bacteria, remove toxic and harmful organic elements from water and air and for self-sterilize glass surfaces [8,9,10,11].

However, it is difficult to separate TiO_2_ NPs after a photochemical reaction, which limits their practical applications [12]. TiO_2_ NPs aggregate easily in solution, which reduces their photocatalytic efficacy because of the decreased surface area. These limitations can be overcome by preparing TiO_2_ nanofibers (NFs) using simple, rapid and cost-effective electrospinning (ES) methods [13,14,15,16,17,18]. TiO_2_ NFs have gained increased attention because of their mesoporous structure [19], stability in solution, little or no aggregation, high surface to volume ratio that enhances photocatalytic reactions and their ease in separation and collection from solution after photochemical reactions [20,21]. However, the photocatalytic efficacy of TiO_2_ NFs is comparatively low and is effective only under ultraviolet (UV) light because of their relatively large band-gap energy and low-ordered crystalline structure [22]. An exceptional feature of TiO_2_ nanoparticles (NPs) is their photocatalytic activity that enhances the bacterial killing when exposed to UV light [7,23]. TiO_2_ NPs tend to exist in three principal forms, namely brookite, rutile and anatase, and it has been reported that the anatase form has a high photocatalytic and antibacterial activity [23,24,25,26]. A major biomedical application of TiO_2_ NPs is to prevent biofilm formation on medical devices that is related to infections and sepsis [3,27,28]. Several researchers have focused on the antibacterial and antibiofilm activities of TiO_2_ NPs under UV light against standard bacterial strains, e.g., ATCC, MTCC and NCIM. However, limited work has been published on the antibacterial and antibiofilm activities of TiO_2_ NFs without application of UV light against drug resistant isolates. The objective of present investigation is to explore the antibacterial and antibiofilm efficacies of TiO_2_ NFs in dark against two major human pathogenic drug resistant bacteria i.e., Gram-positive *methicillin-resistant Staphylococcus aureus* (MRSA) and Gram-negative *Pseudomonas aeruginosa* by using different methods.

## 2. Experimental Methodology

### 2.1. Electrospinning and Heating Protocol

Both the sol-gel and electrospinning approaches were used to synthesize electrospun TiO_2_ NFs. Briefly, Titanium isopropoxide (IV), acetic acid and ethanol were mixed and stirred with respect to volume ratio of 3:1:3. After that, 12% by weight of polyvinylpyrrolidone (PVP) was dissolved in the obtained TiO_2_ solution. This mixed TiO_2_/PVP sol-gel was then placed within a plastic syringe for electrospinning experiment. Additional details are provided in a preliminary study [15]. Thermal gravimetric analysis (TGA) and differential scanning calorimetry (DSC) for the non-isothermal heating of electrospun TiO_2_ NFs were performed on a Mettler Teledo thermal gravimetric analyzer TGA/DSC. The samples were heated from ambient temperature to 900 °C at a rate of 10 °C/min with an argon protective gas of 20 mL/min in various mixtures of air and argon. The thermal experiments were carried out by utilizing alumina crucibles that were charged with 25 mg of sample and in mixtures of 50% air-50% argon, 25% air-75% argon and 100% argon. It is worth noting that the argon shielding gas is included in the relative percentage of air to argon gas. For safety reasons, samples that were contacted with 100% air were heated in an oven under the same conditions [29].

### 2.2. Characterization of Electrospun TiO_2_ NFs

The morphological and structural properties of as-prepared NFs were characterized by SEM (FEI Inspect S50) and TEM (FEI Morgagni 268). The elemental composition was determined by energy-dispersive spectroscopy (EDX). A strong correlation can be established from the initial microstructure images. The TiO_2_ grains were structured as microspheres and a complete description of the microstructure is provided. The microstructure relates to monitoring by three-dimensional imaging of the evolution of internal porosity as a function of annealing temperature. A Jasco V-670 UV–visible diffuse reflectance spectrophotometer (DRS) under a wavelength ranging between 200 and 750 nm was used to estimate the band gap energy (*E_g_*) of various TiO_2_ NFs. 

The values of band gap energy ($E_{g}$) were calculated from the absorption spectra versus wavelength using the following expression:

(1) $$E_{g}=\frac{hC}{\lambda_{0}}$$

In this expression, *h* is Planck’s constant (6.626 × 10^−34^ J.s) and *C* is the speed of light (3 × 10^8^ m/s). $\lambda_{0}$ (expressed in nm) is the cut off wavelength obtained from the absorption spectra [30]. Accordingly, $\lambda_{0}$ denotes the absorption edge wavelength, obtained from the offset wavelength derived and extrapolated from the low energy absorption band. 

### 2.3. Evaluation of Antibacterial Activity of Electrospun TiO_2_ NFs

#### 2.3.1. Bacterial Culture

The laboratory strain of Gram-negative *Pseudomonas aeruginosa* PAO1 and Gram-positive methicillin resistant *Staphylococcus aureus* (MRSA) ATCC 33591 used in this study was obtained from Molecular Microbiology Laboratory, Institute for Research and Medical Consultations, Imam Abdulrahman Bin Faisal University, Dammam, Saudi Arabia. The bacterial strains preserved in glycerol cultures (−80 °C) were cultivated on Tryptic soy broth (TSB) at 37 °C in a shaker incubator before being used for microbial studies.

#### 2.3.2. Investigation of Minimum Inhibitory and Minimum Bactericidal Concentration (MIC/MBC) Values of Electrospun TiO_2_ NFs

The MIC values of TiO_2_ NFs against *P. aeruginosa* and MRSA was estimated by serial two-fold dilutions of TiO_2_ NFs from 32 to 1 mg/mL as described previously [31,32]. The determination of MBC values was also investigated as method described in previous studies [32,33].

### 2.4. Effect of TiO_2_ NFs on Biofilm Formation

The antibiofilm potential of TiO_2_ NFs against *P. aeruginosa* and MRSA biofilm was examined quantitatively in a sterilized 96-well polystyrene (flat bottom) microtiter tissue culture plate using crystal violet assay as described in our previous study [31,33].

### 2.5. Effect of TiO_2_ NFs on the Morphology of P. aeruginosa and MRSA: SEM Analysis

Further, the effects of TiO_2_ NFs on the morphological features of *P. aeruginosa* and *S. aureus* cells were analyzed by SEM. In Brief, ~10^6^ CFU/mL of *P. aeruginosa* and *S. aureus* cells treated with 1 mg/mL of TiO_2_ NFs for 18 h were incubated at 37 °C [33,34]. After incubation, the treated and untreated samples were centrifuged at 10,000 rpm for 15 min. The obtained pellets were washed with PBS (1×) three times and fixed with primary fixative (i.e., 2.5% glutaraldehyde) for 6 h at 4 °C and then further fixed with secondary fixative (i.e., 1% osmium tetroxide) for 1 h. After fixation, the samples were dehydrated by a series of ethanol [34,35]. The cells were then fixed on the aluminum stubs, dried in a desecrator and coated with gold. Finally, the treated and untreated samples were examined by SEM.

## 3. Results and Discussion

### 3.1. Effects of the Calcining Atmosphere on TiO_2_ Colour

**Figure 1:**
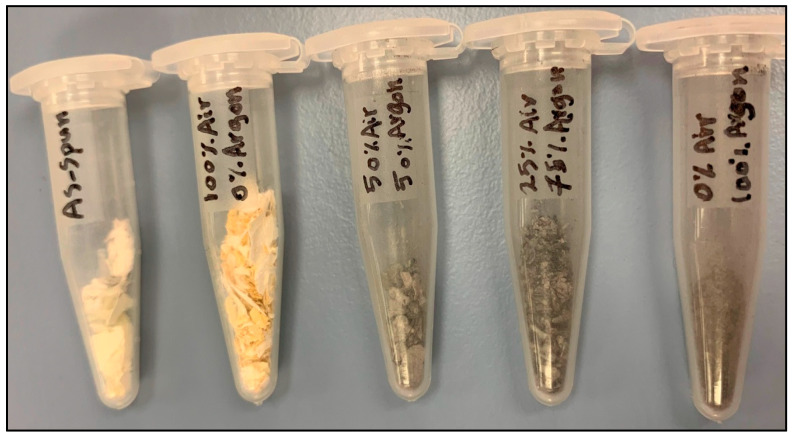
Color changes in electrospun titanium oxide (TiO_2_) nanofibers (NFs) in argon-air mixtures.

Figure 1 presents a gradual color change from white to dark grey after heat treatment in 100% air and in different mediums of air–argon compositions up to 100% argon medium. This change is likely because of oxygen vacancy defects. The change and the intensification of the color are mainly a result of defects associated with oxygen vacancies that rise from an increase in argon content [36].

### 3.2. Microstructure Analysis of the Prepared NFs

**Figure 2:**
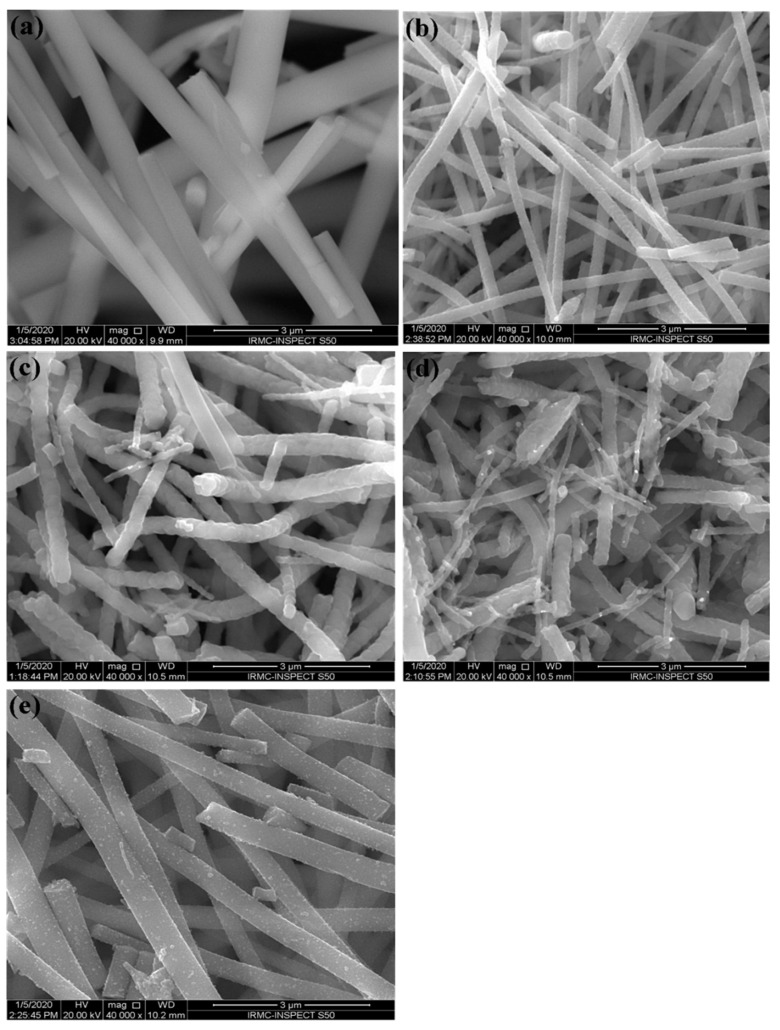
Scanning electron microscopy (SEM) of TiO_2_ NFs calcined in different air and argon mixture. (**a**) As-spun TiO_2_, (**b**) 100% Air, (**c**) 50% Air and 50% Argon, (**d**) 25% Air and 75% Argon and (**e**) 100% Argon.

**Figure 3:**
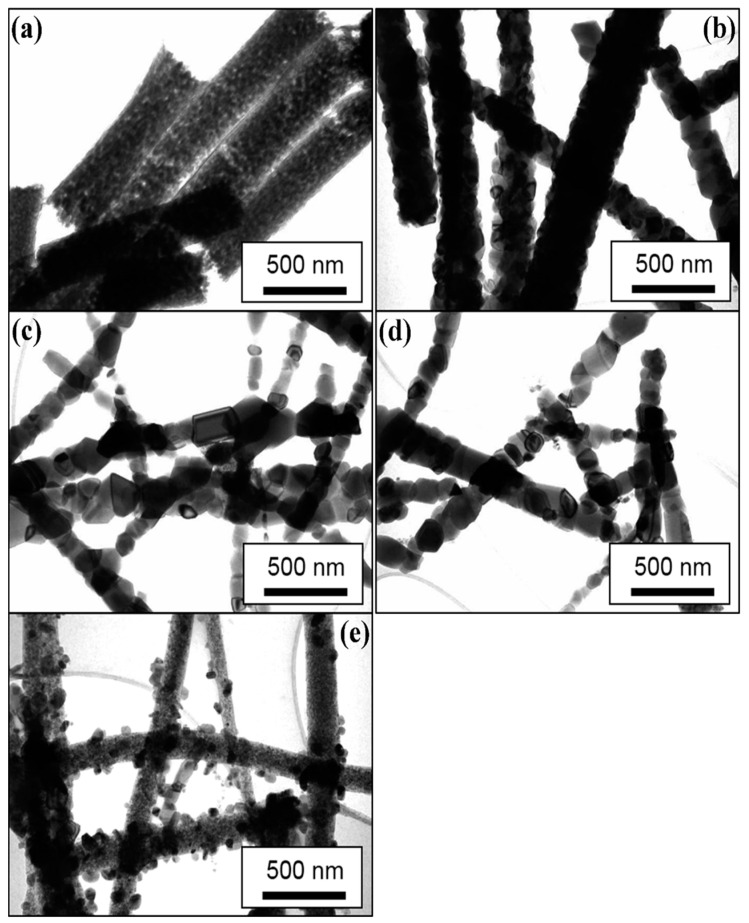
Transmission electron microscopy (TEM) of TiO_2_ NFs calcined in different air and argon mixture. (**a**) As-spun TiO_2_, (**b**) 100% Air, (**c**) 50% Air and 50% Argon, (**d**) 25% Air and 75% Argon and (**e**) 100% Argon.

**Figure 4:**
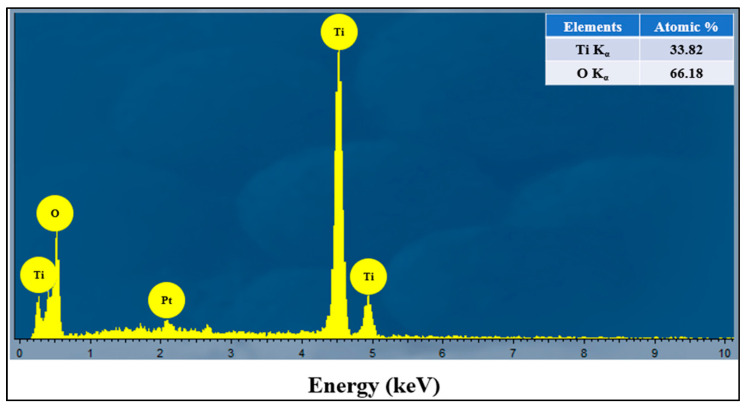
An energy-dispersive X-ray spectroscopy (EDX) spectrum of electrospun TiO_2_ NFs prepared in 50% air-50% argon mixture.

**Figure 5:**
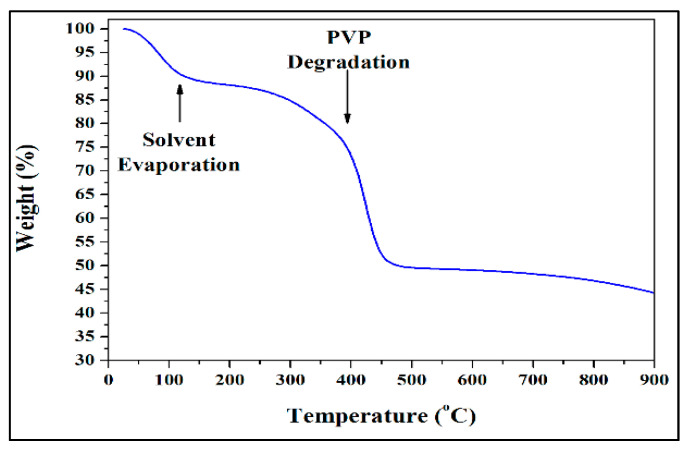
Thermogravimetric analysis (TGA) performed for electrospun TiO_2_ NFs prepared under 50% air-50% argon mixture.

Figure 2 and Figure 3 show the typical SEM and TEM micrographs of the as-spun TiO_2_ and calcined NFs. The electrospinning process could produce good quality TiO_2_ NFs, possibly without nodes and defects. The diameter of the as-spun fibers varied between 80 and 600 nm, whereas the estimated average thickness was ~400 nm (Figure 2a). Upon annealing in different mediums of air/argon (100%-0%, 50%-50%, 25%-75%, and 0%-100%), the fibers shrank, and their morphology changed slightly from smooth to rough. This figure also shows the presence of a heterogeneous matrix made up of agglomerated grains for the initial microstructure and leads to faster granular growth. The fibers size was between 50 and 300 nm (Figure 2b–e). Several thin-fibers of about 50 nm were perceived in specimens annealed under 25-75% air-argon. The quality and shape of fiber mats were preserved after calcination as clarified by the TEM images (Figure 3b–e) unlike the electrospun fibers that are often composed of oxide nanoparticles (Figure 3a) [37]. The as-spun fibers showed organic species, whereas the annealed fibers exhibited a solid morphology with high-quality individual particles in the range of 100 nm. The annealing of TiO_2_ NFs at 900 °C in 50%-50% air-argon led to pure TiO_2_ fibers formation, which was proven by EDX and TGA characterization techniques. In Figure 4, the EDX spectrum illustrates high-intensity O and Ti peaks and a small Pt peak from the platinum coating on the TiO_2_ NFs heated in 50-50% air-argon, which is mainly similar to those observed in specimens annealed in 100% air, 25% air-75% argon, and 100% argon. Figure 5 shows the TGA result for samples heated under 50-50% air-argon medium. The PVP polymer and organic material are completely removed from the electrospun TiO_2_ NFs at ~450 °C, and ~100 °C, respectively.

### 3.3. Wide-Band Gap Analysis of Calcined Electrospun TiO_2_ NFs

**Figure 6:**
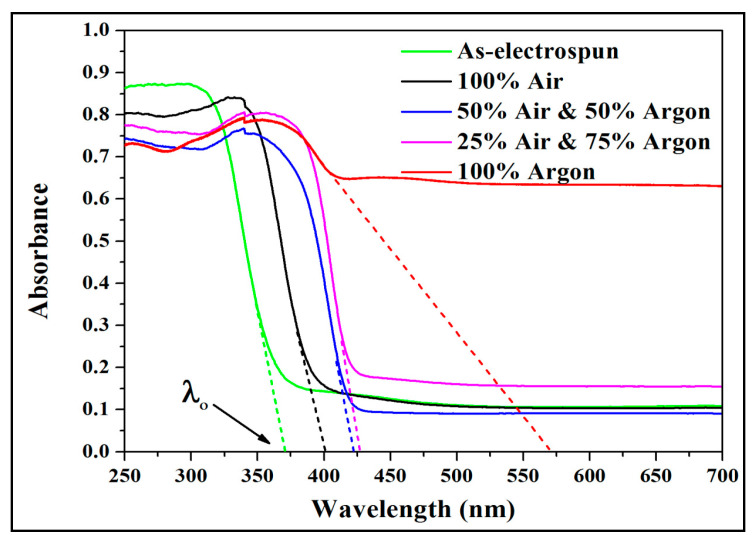
UV-vis diffuse reflectance spectrophotometer (DRS) spectra of electrospun TiO_2_ NFs obtained before calcination and those obtained after calcination in various air-argon media.

Figure 6 shows the UV-vis DRS spectra of as-electrospun TiO_2_ NFs calcinated in air-argon media at 900 °C and cooling to ambient temperature. Table 1 shows the values of band-gaps at room temperature for various TiO_2_ NFs. The band-gap value reduced from 3.33 eV for as-spun and non-calcinated samples to about 3.09 eV for the ones calcinated in 100% air. Under various air-argon environments, the value of *E_g_* decreased from about 3.09 to 2.18 eV with an increase in argon content. A previous study on similar specimens revealed that the growth of vacancies was minimal and the reduction of *E_g_* value was ascribed to the increase in crystallinity [38]. The measured difference agrees with that weighted according to the concentration of pure anatase and rutile phases [38,39,40]. Alterations in levels and phase mixing and gradual development of oxygen vacancies are two factors that can reduce the band-gap energy with argon introduction. The measured energy gap was 2.18 eV for sample heated in 100% argon and for the phase composition for which the difference according to the concentration would be 3.05 eV. The difference of 0.87 eV is assigned to the development of oxygen vacancies and allows a greater density of charge carriers. The development of oxygen vacancies leads to the creation of Ti^3+^ centers or unpaired electrons that generate vacant states under the conduction band [41,42]. The development of oxygen vacancies for different argon concentrations has been previously discussed [38]. When the specimen is annealed in argon, oxygen disappears and the non-stoichiometric anatase (TiO_2−x_) forms [43]. The formation of oxygen vacancy defects in titanium oxide is induced from the occurrence of new localized states of oxygen vacancies between the conduction and valence bands. The excitation of electrons from the valence band to the vacant oxygen states can be done in visible light. With rising argon amount, the effective *E_g_* moves thoroughly to the red region, the specimen is being active under visible light and thus the *E_g_* is reduced. So, the mutual effects of the formation of oxygen vacancies and crystallinity treatment have prolonged the excitation of light of electrospun TiO_2_ NFs from ultraviolet to visible light range without the need of chemical doping.

### 3.4. Antibacterial and Antibiofilm Activity of TiO_2_ NFs

#### 3.4.1. MIC and MBC

The microbiocidal activities of TiO_2_ photocatalysis were reported for the first time by Matsunaga and co-workers in 1985 [44]. They investigated the killing of bacteria and yeast cells in water by employing TiO_2_-Pt photocatalysts in near-ultraviolet radiation. They reported that the inhibition of respiratory activity was the mechanism for cell death.

In this research work, the antibacterial property (MIC/MBC) of TiO_2_ NFs calcined with different ratios of air–argon mixtures (i.e., 100% air, 50% air-50% argon, 25% air-75% argon, and 100% argon) has been investigated against *P. aeruginosa* and MRSA (Supplementary Figure S1). The MIC/MBC values of TiO_2_ NFs heated with different ratios of air-argon mixtures against *P. aeruginosa* and MRSA are presented in Table 2. TiO_2_ NFs heated in the presence of 25% air-75% argon showed a maximum antibacterial activity and MIC/MBC values against *P. aeruginosa* were 3 and 6 mg/mL and for MRSA it was 6 and 12 mg/mL, respectively (Table 2). Based on the MIC and MBC results, it was observed that Gram-negative *P. aeruginosa* was more susceptible to TiO_2_ NFs than Gram-positive MRSA. These results agree with results from previous studies [45,46], and may occur owing to differences in their cell wall structures and to bacterial strain growth rate [45,46,47]. Pigeot-Rémy and co-workers [48] investigated the effects of TiO_2_ particles against *E. coli K-12* in the dark and reported that the attachment of NPs to bacterial surfaces causes membrane damage and perturbation, which may increase the permeability of the outer cell membrane and the resultant damage to the envelope of bacterial cells leads to bacterial cells death.

#### 3.4.2. Effects of Electrospun TiO_2_ NFs on the Morphology of Bacterial Cells

**Figure 7:**
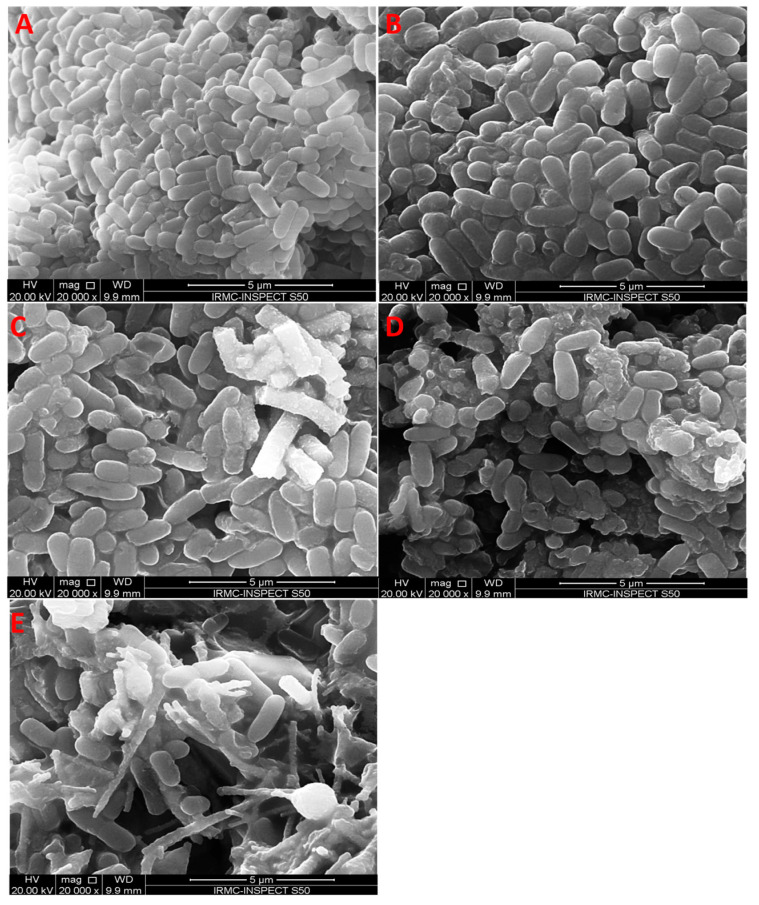
Effect of electrospun TiO_2_ NFs on the morphological aspects of *P. aeruginosa* as examined by scanning electron microscopy: (**A**) control without any treatment and treated with TiO_2_ calcined in (**B**) 100% Air, (**C**) 50% Air and 50% Argon, (**D**) 25% Air and 75% Argon; and (**E**) 100% Argon.

**Figure 8:**
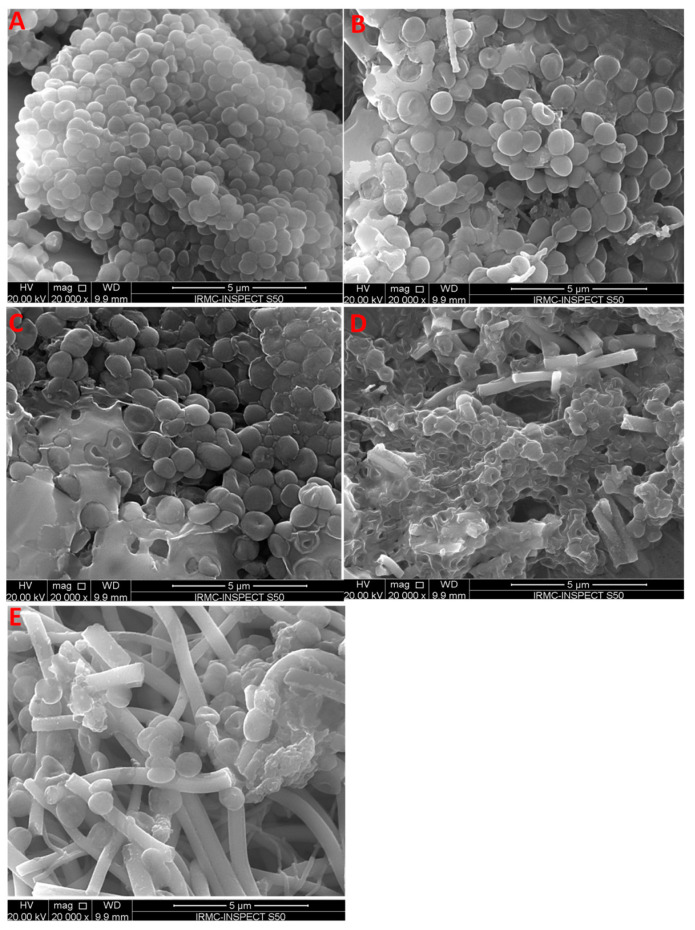
Effect of electrospun TiO_2_ NFs on the morphological aspect of *S. aureus* as examined by scanning electron microscopy: (**A**) control without any treatment and treated with TiO_2_ NFs calcined in (**B**) 100% Air; (**C**) 50% Air and 50% Argon; (**D**) 25% Air and 75% Argon, and (**E**) 100% Argon.

Morphological alterations in Gram-negative P. aeruginosa (Figure 7) and Gram-positive MRSA (Figure 8) after exposure to TiO_2_ NFs were further examined by SEM. The untreated P. aeruginosa had a normal, rod-shaped structure and regular, smooth and intact cell surface (Figure 7). However, the morphology of *P. aeruginosa* cells was altered considerably, and cells were damaged to different extents after treatment with TiO_2_ NFs. After 18 h of treatment, the cell envelope and cell wall were rough, irregular, abnormal in form and main damage was categorized by the creation of “pits” and depressions that probably lead to a loss of bacterial cell membrane integrity (Figure 7). Similarly, the untreated Gram-positive MRSA was normal with smooth and regular cell surfaces (Figure 8). However, MRSA cells treated with TiO_2_ NFs exhibited noticeable alterations and damage and the clusters of NFs were linked and anchored on the surface of bacterial cells (Figure 8). Irregularities, shallows and depressions on the cell envelopes and cell walls of certain MRSA cells suggest that bacterial damage occurred (Figure 8). SEM analysis showed that TiO_2_ NFs were more effective against P. aeruginosa bacterial cells in comparison with MRSA and were severely injured compared with Gram-positive MRSA. The obtained results may be due to morphological dissimilarities in the cell walls of bacteria. Gram-negative bacterial cells display thin layers of peptidoglycan that facilitate the mobility of metal-ion NPs within cells and facilitate the interaction among NPs and walls of bacterial cells. Gram-negative bacteria exhibit a negative charge due to their high content of lipopolysaccharides. This negative charge attracts and interacts with positive metal ions, which may lead to the NP penetration, intracellular damages and protein and DNA destruction [46]. It was suggested that the interaction of TiO_2_ NPs with bacterial cells in the dark caused bacterial membrane integrity destruction, especially of lipopolysaccharides [48]. TiO_2_ NPs form pores in bacterial cell walls and membranes, which increases the permeability and leads to cell death [10]. However, other published work has shown that the contact among metal oxides and bacterial cells provokes oxidation and formation of reactive oxygen groups including O_2_^•–^, ^•^OH, and H_2_O_2_. These free radicals attack bacteria cell walls and alter the membrane integrity and permeability, which leads to bacterial cell death [48,49,50,51]. It has been reported that the destruction of cell envelope by incorporation of TiO_2_ NPs inside the cells damages bacterial DNA and RNA, which could provoke cell death [48]. The antimicrobial activity of TiO_2_ in the absence of photoactivation has been also reported. Nakano and co-worker [51] stated that TiO_2_ deactivates bacterial DNA and enzymes via coordination of electron-donor groups, like hydroxyls, indoles, carbohydrates, amides, and thiols in the absence of light. Pit formation in bacterial cell walls and envelopes that enhanced the permeability lead to bacterial cell death [51,52]. It has been reported that there is proportional relationship between the light and the antimicrobial activity of TiO_2_. Senarathna et al [53] and Lee et al [54] reported that the presence of sunlight enhanced the antimicrobial activity of TiO_2_ against S. aureus might be due to generation of free radicals [53,54].

#### 3.4.3. Inhibition of Biofilm Formation by TiO_2_ NFs

**Figure 9:**
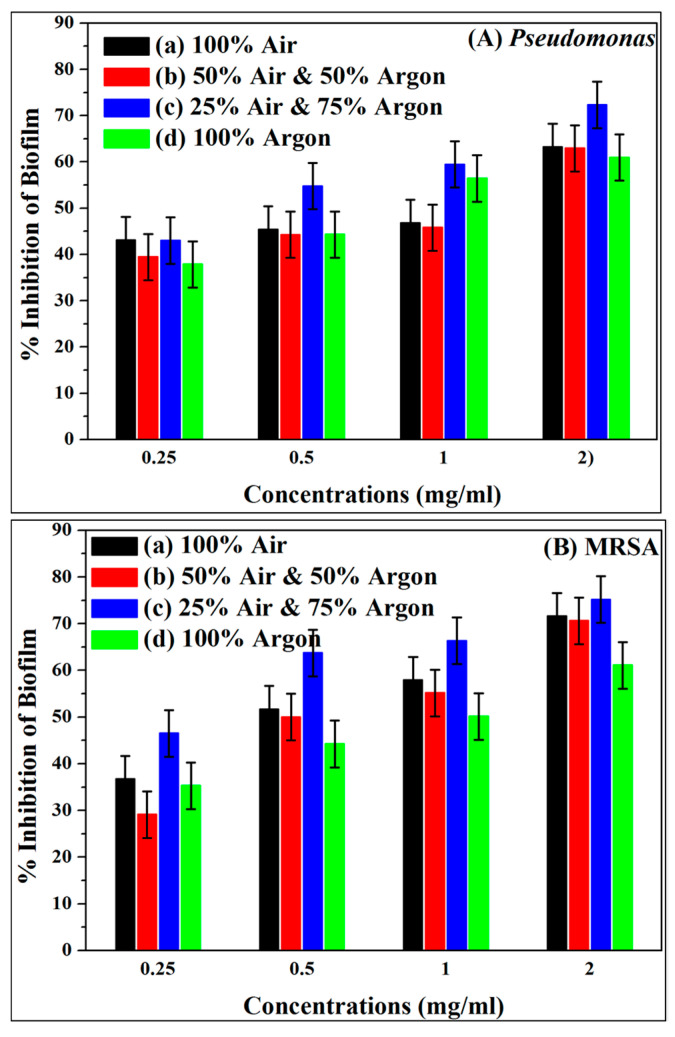
Effect of TiO_2_ NFs calcined in various air–argon environments (a) 100% Air, (b) 50% Air and 50% Argon, (c) 25% Air and 75% Argon, and (d) 100% Argon on biofilm formation abilities of (**A**) *P. aeruginosa* and (**B**) methicillin-resistant *Staphylococcus aureus* (MRSA).

The antibiofilm potential of TiO_2_ NFs heated under different air-argon environments was evaluated at various amounts of 0.25, 0.5, 1.0 and 2.0 mg/mL against MRSA and *P. aeruginosa* biofilms using crystal violet microtiter assays in a 96-well flat-bottom polystyrene plate at OD595 nm. Plots in Figure 9A,B show that TiO_2_ NFs inhibit the biofilms formation by MRSA and *P. aeruginosa* in a dose-dependent manner. It was reported that a rise in TiO_2_ concentration provoked a reduction in the cultivability of bacteria [48]. As shown in Figure 9A,B, TiO_2_ NFs heated in a 25% air-75% argon mixture exhibited the highest biofilm inhibition of about 75.2% for MRSA and 72.3% for *P. aeruginosa,* respectively at 2 mg/mL of TiO_2_ NFs. These results agree with those reported in previous studies [55,56]. In a previous study, epoxy/Ag-TiO_2_ nanocomposites were found to inhibit biofilm creation of *S. aureus* ATCC 6538 and *E. coli K-12* by 67% and 77%, respectively [56].

## 4. Conclusions

This study focuses on the heat treatment of TiO_2_ NFs to develop photoactive titanium photocatalysis in the visible spectrum and to evaluate their antibacterial and antibiofilm potential against Gram-negative bacteria *P. aeruginosa* and Gram-positive MRSA. The *E_g_* value was 3.09 eV for specimens heated in 100% air and 2.18 eV for the ones heated in 100% argon. The value of *E_g_* decreased systematically with rising argon amount in the various air-argon mixtures. The increase in the amount of argon brings the state under the TiO_2_ conduction band. TiO_2_ NFs calcined in a 25% air-75% argon environment showed maximum antibacterial and antibiofilm activities. The MIC/MBC and SEM results show that TiO_2_ NFs were more operative against Gram-negative *P. aeruginosa* than Gram-positive *S. aureus*. The inhibition of biofilm formation by TiO_2_ NFs shows that TiO_2_ NFs inhibit the biofilms formation by MRSA and *P. aeruginosa* in a dose-dependent manner. From the obtained data on antibacterial antibiofilm analysis, it has been concluded and suggested that TiO_2_ NFs can be used in hydrophilic coatings, coating of various inanimate object surfaces, such as metals, glass, medical devices and equipment to prevent biofilm formation on medical devices or medical device-related infections and sepsis, and also can be applied in food packaging, wastewater treatment and purification, self-cleaning and self-disinfecting, killing of bacteria and the removal of toxic and damaging organic compounds from water and air.

## Figures and Tables

**Table 1:** Band gap energies for as-electrospun TiO_2_ nanofibers (non-calcinated), and TiO_2_ NFs obtained after calcination at 900 °C in various air-argon media.

Calcination Conditions	*E_g_* (eV)
As-electrospun	3.33
100% Air	3.09
50% Air and 50% Argon	2.94
25% Air and75% Argon	2.91
100% Argon	2.18

**Table 2:** Minimum inhibitory concentration (MIC) and minimum bactericidal concentration (MBC) (mg/mL) values of tested electrospun TiO_2_ nanofibers against Methicillin resistant *S. aureus* and *P. aeruginosa*.

Electrospun TiO_2_ Nanofibers Code	Calcination Conditions	Methicillin Resistant *S. aureus*		*P. aeruginosa*	
		MIC	MBC	MIC	MBC
(a)	100% Air	7	14	7	14
(b)	50% Air and 50% Argon	7	14	7	14
(c)	25% Air and 75% Argon	6	12	3	6
(d)	100% Argon	>16	>32	>16	>32

## Supplementary Materials

The following are available online at https://www.mdpi.com/2079-6382/9/9/572/s1, Figure S1: represents MHA plates showing MBC values of tested electrospun TiO_2_ NFs in various air–argon environments.

## References

[1] Fujishima A., Rao T.N., Tryk D.A. (2000). Titanium dioxide photocatalysis. J. Photochem. Photobiol. C: Photochem. Rev..

[2] Mills A., Le Hunte S. (1997). An overview of semiconductor photocatalysis. J. Photochem. Photobiol. A: Chem..

[3] Gupta S.M., Tripathi M. (2011). A review of TiO2 nanoparticles. Chin. Sci. Bull..

[4] Kamat P. (2002). Photophysical, Photochemical and Photocatalytic Aspects of Metal Nanoparticles. J. Phys. Chem. B.

[5] Mantravadi H.B. (2017). Effectivity of Titanium Oxide Based Nano Particles on E. coli from Clinical Samples. J. Clin. Diagn. Res..

[6] Awati P., Awate S., Shah P., Ramaswamy V. (2003). Photocatalytic decomposition of methylene blue using nanocrystalline anatase titania prepared by ultrasonic technique. Catal. Commun..

[7] Othman S., Salam N.R.A., Zainal N., Basha R.K., Talib R. (2014). Antimicrobial Activity of TiO2Nanoparticle-Coated Film for Potential Food Packaging Applications. Int. J. Photoenergy.

[8] Hashimoto K., Irie H., Fujishima A. (2005). TiO2Photocatalysis: A Historical Overview and Future Prospects. Jpn. J. Appl. Phys..

[9] Fujishima A., Zhang X., Tryk D.A. (2008). TiO2 photocatalysis and related surface phenomena. Surf. Sci. Rep..

[10] Haghi M., Hekmatafshar M., Janipour M.B., Gholizadeh S.S., Faraz M.K., Sayyadifar F., Ghaedi M. (2012). Antibacterial effect of TiO2 nanoparticles on pathogenic strain of E. coli. Int. J. Adv. Biotechnol. Res..

[11] Runa S., Khanal D., Kemp M.L., Payne C.K. (2016). TiO2 Nanoparticles Alter the Expression of Peroxiredoxin Antioxidant Genes. J. Phys. Chem. C.

[12] Schmidt H., Naumann M., Muller T., Akarsu M. (2006). Application of spray techniques for new photocatalytic gradient coatings on plastics. Thin Solid Film..

[13] E Teo W., Ramakrishna S. (2006). A review on electrospinning design and nanofibre assemblies. Nanotechnology.

[14] Albetran H., Dong Y., Low I.M. (2015). Characterization and optimization of electrospun TiO2/PVP nanofibers using Taguchi design of experiment method. J. Asian Ceram. Soc..

[15] Albetran H., Haroosh H., Dong Y., Prida V.M., O’Connor B.H., Low I.M. (2014). Phase transformations and crystallization kinetics in electrospun TiO2 nanofibers in air and argon atmospheres. Appl. Phys. A.

[16] Albetran H., Low I.M. (2016). Crystallization kinetics and phase transformations in aluminum ion-implanted electrospun TiO2 nanofibers. Appl. Phys. A.

[17] Albetran H., O’Connor B.H., Prida V.M., Low I.M. (2015). Effect of vanadium ion implantation on the crystallization kinetics and phase transformation of electrospun TiO2 nanofibers. Appl. Phys. A.

[18] Albetran H., Low I. (2019). Parameters controlling the crystallization kinetics of nanostructured TiO2—An overview. Mater. Today Proc..

[19] Zhang X.-W., Xu S., Han G. (2009). Fabrication and photocatalytic activity of TiO2 nanofiber membrane. Mater. Lett..

[20] Chandrasekar R., Zhang L., Howe J.Y., Hedin N.E., Zhang Y., Fong H. (2009). Fabrication and characterization of electrospun titania nanofibers. J. Mater. Sci..

[21] Li Q., Satur D.J.G., Kim H., Kim H.G. (2012). Preparation of sol–gel modified electrospun TiO2 nanofibers for improved photocatalytic decomposition of ethylene. Mater. Lett..

[22] Xu S., Li A., Poirier G., Yao N. (2012). In Situ Mechanical and Electrical Characterization of Individual TiO2Nanofibers Using a Nanomanipulator System. Scanning.

[23] Ahmad R., Sardar M. (2013). TiO2 nanoparticles as an antibacterial agents against E. coli. Int. J. Innov. Res. Sci. Eng. Technol..

[24] Albetran H., Low I. (2020). Crystallization kinetics study of In-doped and (In-Cr) co-doped TiO2 nanopowders using in-situ high-temperature synchrotron radiation diffraction. Arab. J. Chem..

[25] Albetran H., Vega V., Prida V.M., Low I.-M. (2018). Dynamic Diffraction Studies on the Crystallization, Phase Transformation, and Activation Energies in Anodized Titania Nanotubes. Nanomaterials.

[26] Albetran H., Low I.-M. (2016). Effect of indium ion implantation on crystallization kinetics and phase transformation of anodized titania nanotubes using in-situ high-temperature radiation diffraction. J. Mater. Res..

[27] Ravishankar R.V., Jamuna B.A., Mendez-Vilas A. (2011). Nanoparticles and their potential application as antimicrobials. Science against Microbial Pathogens: Communicating Current Research and Technological Advances.

[28] Arora H., Doty C., Yuan Y., Boyle J., Petras K., Rabatic B., Paunesku T., Woloschak G.E., Kumar C.S.S.R. (2010). Titanium Dioxide Nanocomposites. Nanomaterials for Life Sciences.

[29] Albetran H., O’Connor B., Low I.-M. (2016). Effect of calcination on band gaps for electrospun titania nanofibers heated in air–argon mixtures. Mater. Des..

[30] Bhadwal A.S., Tripathi R., Gupta R.K., Kumar N., Singh R.P., Shrivastav A. (2014). Biogenic synthesis and photocatalytic activity of CdS nanoparticles. RSC Adv..

[31] Balasamy R.J., Ravinayagam V., Alomari M., Ansari M.A., Almofty S.A., Rehman S., Dafalla H., Marimuthu P.R., Akhtar S., Hamad M. (2019). Cisplatin delivery, anticancer and antibacterial properties of Fe/SBA-16/ZIF-8 nanocomposite. RSC Adv..

[32] Sultan A., Khan H.M., Malik A., Ansari A., Azam A., Perween N. (2015). Antibacterial activity of ZnO nanoparticles against ESBL and Amp-C producing gram negative isolates from superficial wound infections. Int. J. Curr. Microbiol. App. Sci..

[33] Baig U., Ansari M.A., Gondal M., Akhtard S., Khan F.A., Falathaf W.S. (2020). Single step production of high-purity copper oxide-titanium dioxide nanocomposites and their effective antibacterial and anti-biofilm activity against drug-resistant bacteria. Mater. Sci. Eng. C.

[34] Ali F.A.A., Alam J., Shukla A.K., Alhoshan M., Ansari M.A., Al-Masry W.A., Rehman S., Alam M. (2019). Evaluation of antibacterial and antifouling properties of silver-loaded GO polysulfone nanocomposite membrane against Escherichia coli, Staphylococcus aureus, and BSA protein. React. Funct. Polym..

[35] Shukla A.K., Alam J., Ansari M.A., Alhoshan M., Alam M., Kaushik A. (2019). Selective ion removal and antibacterial activity of silver-doped multi-walled carbon nanotube/polyphenylsulfone nanocomposite membranes. Mater. Chem. Phys..

[36] Gamboa J.A., Pasquevich D.M. (1992). Effect of Chlorine Atmosphere on the Anatase-Rutile Transformation. J. Am. Ceram. Soc..

[37] Szilágyi I.M., Santala E., Heikkilä M., Pore V., Kemell M., Nikitin T., Teucher G., Firkala T., Khriachtchev L., Rasanen M. (2013). Photocatalytic properties of WO3/TiO2 core/shell nanofibers prepared by electrospinning and atomic layer deposition. Chem. Vap. Depos..

[38] Albetran H., O’Connor B.H., Low I.-M. (2016). Activation energies for phase transformations in electrospun titania nanofibers: Comparing the influence of argon and air atmospheres. Appl. Phys. A.

[39] Scanlon D.O., Dunnill C.W., Buckeridge J., Shevlin S.A., Logsdail A.J., Woodley S.M., Catlow C.R.A., Powell M.J., Palgrave R.G., Parkin I.P. (2013). Band alignment of rutile and anatase TiO2. Nat. Mater..

[40] Natoli A., Cabeza A., Torre, Ángeles G., Aranda M.A.G., Santacruz I., Cabeza A. (2011). Colloidal Processing of Macroporous TiO2 Materials for Photocatalytic Water Treatment. J. Am. Ceram. Soc..

[41] Nakamura I., Negishi N., Kutsuna S., Ihara T., Sugihara S., Takeuchi K. (2000). Role of oxygen vacancy in the plasma-treated TiO2 photocatalyst with visible light activity for NO removal. J. Mol. Catal. A: Chem..

[42] Seo H., Baker L.R., Hervier A., Kim J., Whitten J.L., Somorjai G.A. (2011). Generation of Highly n-Type Titanium Oxide Using Plasma Fluorine Insertion. Nano Lett..

[43] Andersson S., Collén B., Kuylenstierna U., Magnéli A., Pestmalis H., Åsbrink S. (1957). Phase Analysis Studies on the Titanium-Oxygen System. Acta Chem. Scand..

[44] Matsunaga T., Tomoda R., Nakajima T., Wake H. (1985). Photoelectrochemical sterilization of microbial cells by semiconductor powders. FEMS Microbiol. Lett..

[45] Nagalakshmi M., Karthikeyan C., Anusuya N., Brundha C., Basu M.J., Karuppuchamy S. (2017). Synthesis of TiO2 nanofiber for photocatalytic and antibacterial applications. J. Mater. Sci. Mater. Electron..

[46] Azizi-Lalabadi M., Ehsani A., Divband B., Alizadeh-Sani M. (2019). Antimicrobial activity of Titanium dioxide and Zinc oxide nanoparticles supported in 4A zeolite and evaluation the morphological characteristic. Sci. Rep..

[47] Vardanyan Z., Gevorkyan V., Ananyan M., Vardapetyan H., Trchounian A. (2015). Effects of various heavy metal nanoparticles on Enterococcus hirae and Escherichia coli growth and proton-coupled membrane transport. J. Nanobiotechnol..

[48] Pigeot-Remy S., Simonet F., Errazuriz-Cerda E., Lazzaroni J., Atlan D., Guillard C. (2011). Photocatalysis and disinfection of water: Identification of potential bacterial targets. Appl. Catal. B: Environ..

[49] Ranjan S., Ramalingam C. (2016). Titanium dioxide nanoparticles induce bacterial membrane rupture by reactive oxygen species generation. Environ. Chem. Lett..

[50] Wang D., Zhao L., Ma H., Zhang H., Guo L.-H. (2017). Quantitative Analysis of Reactive Oxygen Species Photogenerated on Metal Oxide Nanoparticles and Their Bacteria Toxicity: The Role of Superoxide Radicals. Environ. Sci. Technol..

[51] Chakra C.S., Mateti S. (2018). Structural, Antimicrobial and Electrochemical Properties of Cu/TiO2 Nanocomposites. J. Nanosci. Technol..

[52] Nakano R., Hara M., Ishiguro H., Yao Y., Ochiai T., Nakata K., Murakami T., Kajioka J., Sunada K., Hashimoto K. (2013). Broad Spectrum Microbicidal Activity of Photocatalysis by TiO2. Catalysts.

[53] Senarathna U.L.N.H., Fernando N., Gunasekara C., Weerasekera M.M., Hewageegana H.G.S.P., Arachchi N.D., Siriwardena H.D., Jayaweera P.M. (2017). Enhanced antibacterial activity of TiO2 nanoparticle surface modified with Garcinia zeylanica extract. Chem. Cent. J..

[54] Lee W.S., Park Y.-S., Cho Y.-K. (2015). Significantly enhanced antibacterial activity of TiO 2 nanofibers with hierarchical nanostructures and controlled crystallinity. Analyst.

[55] Alavi M., Karimi N., Valadbeigi T., Valadbaeigi T. (2019). Antibacterial, Antibiofilm, Antiquorum Sensing, Antimotility, and Antioxidant Activities of Green Fabricated Ag, Cu, TiO2, ZnO, and Fe3O4 NPs via Protoparmeliopsis muralis Lichen Aqueous Extract against Multi-Drug-Resistant Bacteria. ACS Biomater. Sci. Eng..

[56] Santhosh S.M., Natarajan K. (2015). Antibiofilm Activity of Epoxy/Ag-TiO2 Polymer Nanocomposite Coatings against Staphylococcus Aureus and Escherichia Coli. Coatings.

