# Supplementary material for: Synthesis of Electrospun TiO2 Nanofibers and Characterization of Their Antibacterial and Antibiofilm Potential against Gram-Positive and Gram-Negative Bacteria
Source: Antibiotics (Basel). 2020 Sep 3;9(9):572. doi: 10.3390/antibiotics9090572 (PMC7557960; doi:10.3390/antibiotics9090572)
Supplement: Supplementary file 1 [file antibiotics-09-00572-s001.pdf]

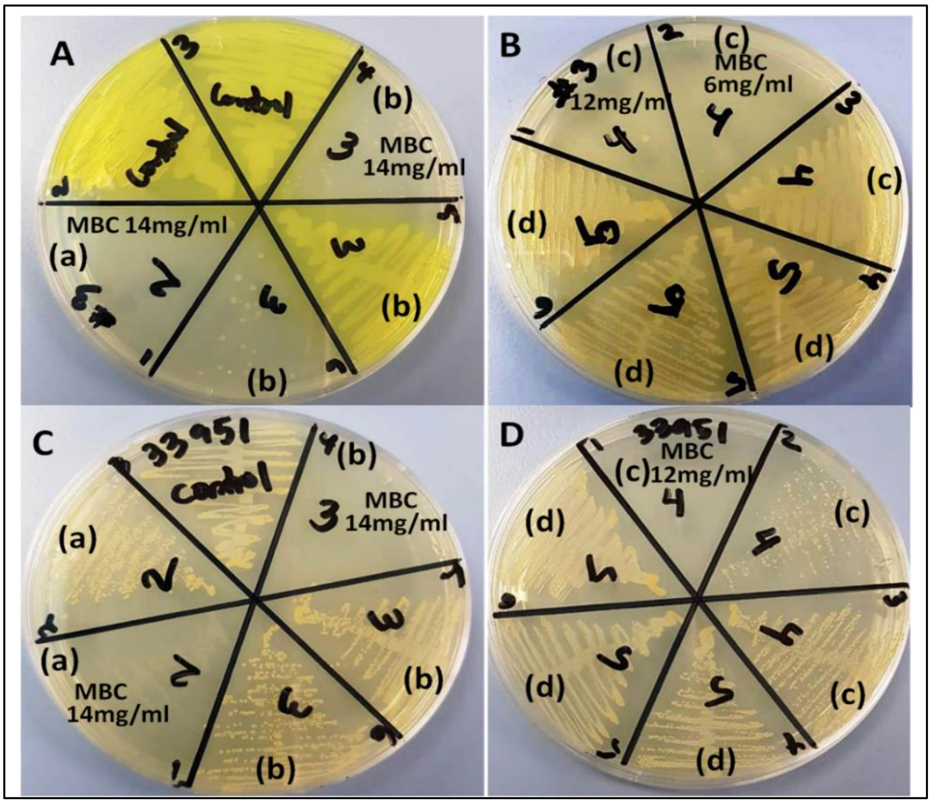

Figure S1. MHA plates showing MBC values of tested electrospun TiO_2 NFs in various air-argon environments of (a) 100% Air, (b) 50% Air & 50% Argon, (c) 25% air & 50% Argon, and (d) 100% Argon against multidrug-resistant *P. aeruginosa* (A & B) and methicillin-resistant *S. aureus* (C & D).
